# Prevalence of HIV among victims of sexual assault who were mentally impaired children (5 to 18 years) in the Mthatha area of South Africa

**DOI:** 10.4102/phcfm.v1i1.5

**Published:** 2009-04-24

**Authors:** Banwari Meel

**Affiliations:** 1 Department of Forensic Medicine, Walter Sisulu University, South Africa

**Keywords:** HIV, rape, child abuse, mental impairment, sexual violence

## Abstract

**Background:**

Protection of children has been identified as a priority in South Africa. Despite a commitment to uphold children's rights, much still needs to be done for the safety of mentally impaired children.

**Method:**

This is a record review of attendees at the Sinawe Centre from 2001 to 2005. It is the only centre in the Mthatha area that provides care for sexually assaulted persons and it is affiliated to the Nelson Mandela Academic Hospital. All mentally impaired victims of sexual assault were recorded on the register.

**Results:**

During the study period, 1,268 individuals, of whom 32 were profoundly mentally impaired, attended the Sinawe Centre following sexual assault. Of these mentally impaired individuals, 28 (87.5%) were below the age of 18 years. Two were males while the rest were females, giving a male to female ratio of 1:15. A close relative was implicated in 29 (90.6%) of the cases. Among the victims were six (18.7%) epileptics who were on treatment. One was 13 years old and pregnant. Four were HIV positive on screening.

**Conclusion:**

Over 2% of the sexual assault victims attending the Sinawe Centre were mentally impaired. Of these, 12.5% were HIV seropositive.

## INTRODUCTION

Sexual violence is ubiquitous; it occurs in every culture, at all levels of society and in every country in the world. Data from studies across the world indicate that, in some parts of the world at least, one woman in every five has suffered an attempted or completed rape.^1^ Most of the time, females are the victims of sexual assault but it does occur to males too, particularly in prisons and hostels. Mentally impaired children are more vulnerable to sexual assault in poverty-stricken areas such as Mthatha. Victims of child sexual abuses are very likely to experience depression.^1^ About 235,000 children in Angola are born mentally impaired every year, followed by South Africa with 160,000 and Malawi with 115,000.^2^ Children with mental disabilities are the weakest in terms of negotiation of safe sex in society and are therefore vulnerable to sexual abuse.

In South Africa, children (0–18 years) constitute 43% (19 million) of the population.^3^ Two-thirds of these children live in poverty and are highly susceptible to the consequences of poverty, such as physical and sexual abuse.^4^ In 2002, alone 21,000 child rapes were reported in South Africa. According to the South African Police, only one in 35 rapes is actually reported.^5^ Therefore, the real incidence of rape may well be in excess of a million per year. The actual incidence of child sexual abuse has reached alarming proportions, which should signal urgent intervention from the highest levels of government and society at large.^5^ A recent study on the prevalence of rape in the Transkei area showed that 68% of children under 20 years of age had been sexually assaulted. Of these, 34% were less than 10 years old and 7.2% were less than five years old.^6^

The cases of rape reported to the police (240 incidents per 100,000 per year) represent the tip of the iceberg of sexual coercion. Forced sexual initiation is reported by almost a third of adolescent girls. In addition, coerced sex is a common problem in schools, in workplaces and amongst peers.^7^

South Africa is home to many children living in absolute poverty. Using R1200 ($120) per household per month as the poverty line, it has been calculated that two-thirds of South African children are living below the poverty line. Some 4.5 million children were reported as sometimes, often or always going to bed hungry because there was not enough food in the house. Poverty is being exacerbated by an unchecked HIV/AIDS pandemic that affects poor communities disproportionately and, in turn, deepens poverty.^8^ Appropriate responses to children in the face of the HIV/AIDS pandemic are critical. In 2008, about 5.7 million people were estimated to be living with HIV in South Africa, with 18.1% of these belonging to between 15 and 49 years of age group. A total of 280,000 children aged 0–14 were living with HIV, as were 3,200,000 women aged 15 and up.^9^

HIV infection and psychiatric disorders have a complex relationship. HIV infection could lead to psychiatric disorders, and psychiatric patients are more vulnerable to HIV infection.^10^ Many mentally ill people already have or may become infected with HIV due to a range of factors, including lack of information and poor risk prevention skills.^11^ The purpose of this report is to highlight the magnitude of HIV and mental impairment among sexually assaulted children in the Mthatha region of South Africa.

## METHOD

This is a record review of mentally impaired victims of sexual assault who presented at the Sinawe Centre from January 2001 to December 2005. Entries included the names, addresses, age and mental status of the victims. The data of those below 18 years of age were analysed. Sinawe is the only centre that caters to Mthatha and the adjoining districts such as Tsolo, Qumbu, Mquanduli, Ngqeleni and Libode and covers a population of about 400,000. This centre provides 24-hour services. There are 15 staff members, including medical officers trained in psychiatric assessment. Psychiatric trained nursing staff, social workers, psychologists and police officers are part of the 15 staff members. A mentally impaired person was someone who did not reply to questions rationally and whose parents or guardians had reported the deficient mental state of the victim since birth. The nurses trained in psychiatry identified these victims. HIV screening was done by the trained nurses by using a rapid test and confirmed by Elisa tests in the laboratory.

## RESULTS

There were 32 cases of mentally impaired persons who attended Sinawe Centre during the five-year period from 2001 to 2005. Of these, 28 (87.5%) were below the age of 18 years. Two were male while the rest were female, giving a male to female ratio of 1:15. A close relative was implicated in 29 (90.6%) of the cases. There were six (18.7%) epileptics who had been on treatment for more than six months and whose seizures were controlled through treatment. One of the victims was 13 years old and pregnant but not an epileptic. Four were HIV positive according to the screening test.

## DISCUSSION

There is a scarcity of literature on the problem of sexual assault among mentally impaired children. It is also difficult to answer how much rape occurs in South Africa. It is even more difficult to answer how many mentally impaired persons are sexually abused in South Africa. The mentally impaired are generally weaker and voiceless people in a community whereby their problem is usually ignored by the community at large.

Sexual violence has a significant impact on the health of the population. The potential reproductive and sexual health consequences of sexual assault are numerous, such as unwanted pregnancies and sexually transmitted infections (STIs), including HIV/AIDS. Illegitimate pregnancies become even more problematic when the mother is mentally impaired. A mentally impaired person will delay in accessing Termination of Pregnancy (TOP)’ services because of her ignorance. The mental health consequences of sexual violence can be just as serious and long lasting.^1^ Life-threatening situations such as suicide are also not uncommon.

Crime against children has increased by a staggering 45% over the past three years. A total of 85,000 cases were reported nationally last year, which is about 235 crimes a day. This was substantially higher than the 59,526 cases reported the previous year.^12^ Among the mentally impaired victims in this study, there were 28 (87.5%) children under the age of 18 years. Twenty-nine (90.6%) of the 32 mentally impaired victims knew their perpetrators. Most of the time, they were family members, the protectors becoming the perpetrators.

It is difficult to obtain an accurate history from the mentally impaired and they are difficult to examine as well. Because they cannot communicate their predicament effectively, they are often repeatedly abused. There are no safe places for these people in a poverty-stricken society. Epilepsy was common among the mentally impaired victims in this study – six (18.7%) epileptics were on treatment. One of the epileptic victims was abused when she went to the health centre for her treatment. She had been raped before and the police had been informed but no action was taken to prevent recurrences. Perpetrators of sexual assault identify their vulnerable victims, the place and time to commit the crime. In many instances, the victims are invited to the perpetrators’ homes at night and at times, the victims’ own houses are visited when the parents or guardians are away.

Like HIV/AIDS, sexual assault carries a great stigma in the Xhosa community. Much anecdotal evidence exists that suggests that the stigmatising experiences of both HIV-positive individuals and survivors of sexual violence are similar.^10^ Both groups are often discredited or viewed as ‘spoilt’ and are usually held personally responsible for their disease or the sexual assault.^13^ Generally, they do not want to disclose this secret to other people in the community, especially when the perpetrator is either a breadwinner or a very close relative. This becomes a problem when the victim becomes pregnant following the sexual assault.

HIV/AIDS is increasing among the victims of sexual assault. A recent study showed that the rate of sexual assault runs parallel with HIV prevalence in the Transkei region.^10^ Poverty, sexual assault and HIV are all interrelated. When all of these are added to mental retardation, the problem becomes huge. It is difficult to study the number of seroconversions among mentally impaired victims, as there are a high number of defaulters of post-exposure prophylaxis.

Not a single reported study has been done on review cases of sexual assault. Some of the victims even refuse to consent to a physical examination. Most research focus on estimating the number of people who hold stigmatising attitudes, but of greater importance is the need to understand the impact of stigma and discrimination on public health goals, for example, voluntary counselling testing (VTC), uptake of anti-retroviral (ARV) treatment^9^ and the adherence of post-exposure prophylaxis (PEP) following a sexual assault.

This report should serve as a stimulus for a larger study related to sexual assault among the mentally impaired. In the face of the rising HIV/AIDS pandemic, there is a need to deal with sexual assault cases more seriously so that HIV/AIDS can be prevented in the community. Vulnerable members of society must be protected, and perpetrators must be punished for their deeds.
